# Melissopalynological, Physicochemical, Mineral, and Volatile Characterization of *Castanea sativa* and *Myosotis* Honeys in Comparison with Multifloral Honeys

**DOI:** 10.3390/foods15152593

**Published:** 2026-07-24

**Authors:** Esra Demir Kanbur

**Affiliations:** Central Research Laboratory, Application and Research Center, Recep Tayyip Erdoğan University, 53020 Rize, Türkiye; esra.demir@erdogan.edu.tr

**Keywords:** melissopalynology, volatile compounds, mineral composition, monofloral honey, *Castanea sativa* honey, *Myosotis* honey

## Abstract

In this study, honey samples collected from the Varda Plateau (İkizdere Valley, Rize) and Yusufeli regions of northeastern Türkiye were comprehensively characterized in terms of their melissopalynological, physicochemical, elemental, and volatile properties. Ten honey samples collected from different apiaries were analyzed. Melissopalynological results identified two samples as monofloral *Castanea sativa* (chestnut) honeys and three samples as *Myosotis*-dominant honeys, while the remaining samples were classified as multifloral. The pollen spectra reflected the rich floristic diversity of the region, shaped by both forest vegetation and high-altitude herbaceous flora. Moisture and proline analyses indicated that the investigated samples were consistent with the characteristics of natural and well-matured honeys. Volatile compound analysis by HS-SPME/GC–MS revealed substantial qualitative and quantitative differences among samples, with chestnut and *Myosotis* honeys generally exhibiting more distinctive aroma profiles than multifloral honeys. Benzaldehyde, furfural, nonane, and linalool derivatives were among the most frequently detected volatile compounds, although their relative abundances varied considerably among samples. Elemental analysis identified potassium as the predominant mineral in all honey samples, while significant variability was observed for Fe, Zn, Mn, Ni, and Cu concentrations. Particularly, the Yusufeli samples were characterized by markedly higher Fe and Cu contents than the Varda samples, indicating a strong geographical influence on elemental composition. Multivariate analyses, including PCA, HCA, and UpSet analysis, confirmed considerable compositional heterogeneity and demonstrated that both botanical and geographical origins contribute to honey differentiation. Overall, the findings provide new insights into the chemical and botanical characteristics of chestnut, *Myosotis*, and multifloral honeys from the Eastern Black Sea region and highlight the value of integrated analytical and chemometric approaches for honey characterization, classification, and origin assessment.

## 1. Introduction

Honey is a complex natural product whose physicochemical composition, mineral profile, and volatile constituents are strongly shaped by botanical origin, geographical conditions, and environmental factors. These variables not only influence the nutritional and sensory properties of honey but also determine its authenticity and market value. Consequently, the identification of reliable compositional markers linked to floral origin has become a central focus in honey research, particularly in the context of geographical indication and quality control [1]. Recent studies have shown that combining DNA metabarcoding with traditional melissopalynological analysis significantly improves the accuracy of botanical origin determination in honey [2].

Among the available approaches, melissopalynological analysis remains one of the most robust methods for determining the botanical origin of honey, as it directly reflects the pollen spectrum of nectar sources. However, relying solely on pollen analysis may not fully capture the compositional complexity of honey. Therefore, recent studies increasingly emphasize the integration of complementary analytical techniques, including mineral profiling and volatile compound analysis, to achieve a more comprehensive characterization [3].

Mineral composition provides valuable information regarding the geochemical environment of honey production, as elemental content is primarily derived from soil–plant interactions and environmental conditions. Similarly, volatile compounds play a crucial role in defining the sensory identity of honey and are closely associated with floral origin. The combined evaluation of these parameters has been shown to enhance the discrimination of monofloral and multifloral honeys, offering a multidimensional approach to honey authentication [4].

Studies on Turkish honeys have shown that mineral profiles can serve as reliable markers for regional differentiation and authenticity assessment, particularly for honeys produced in mountainous and less industrialized areas [4].

Despite the growing body of research on regional honeys in Türkiye, comprehensive studies integrating melissopalynological, physicochemical, mineral, and volatile analyses remain limited for several ecologically rich highland areas. In particular, the İkizdere Valley, located in the Eastern Black Sea region, represents a biologically diverse yet underexplored environment with significant potential for the production of distinctive highland honeys. The lack of integrated compositional data from this region constitutes a clear gap in the literature [5].

The Eastern Black Sea region of Türkiye is characterized by high precipitation, rugged topography, and exceptional botanical diversity, creating favorable conditions for the production of distinctive highland honeys. Recent studies have highlighted the advantages of DNA-based approaches, such as ITS-based metabarcoding, for improving the accuracy of floral authentication in honey. Compared to traditional melissopalynological analysis, these molecular techniques can provide higher taxonomic resolution and reduce potential biases associated with pollen identification. In this context, the present study focuses on compositional characterization rather than definitive authentication. Previous studies on Ayder, Anzer, and other regional honeys have revealed unique pollen compositions and chemical profiles associated with local flora and altitude [6].

Based on this background, the present study explores whether honeys produced in the İkizdere Valley exhibit distinct compositional signatures that can be directly linked to their botanical origin. To address this hypothesis, an integrated analytical approach combining melissopalynological analysis, physicochemical characterization, mineral profiling, and volatile compound analysis was employed. In addition, multivariate statistical techniques were applied to evaluate whether monofloral and multifloral honeys can be effectively differentiated based on their chemical and aromatic profiles. Although multiple chestnut and *Myosotis* honeys were included, the number of samples within each botanical category remains limited, which should be considered when interpreting the statistical analyses.

## 2. Materials and Methods

### 2.1. Honey Samples

**Figure 1:**
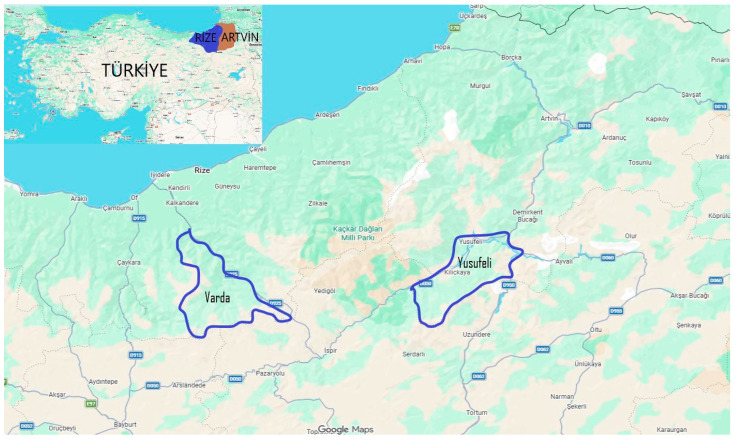
Study area.

A total of ten honey samples were collected from different apiaries located in the Varda Plateau (İkizdere Valley, Rize) and Yusufeli (Artvin), northeastern Türkiye (Figure 1). The sampling sites represent distinct ecological and geographical environments characterized by varying altitudes, climatic conditions, and vegetation structures. Six samples originated from the Varda Plateau, while four samples were collected from the Yusufeli region, allowing the evaluation of both botanical and geographical influences on honey composition. All samples were transported to the laboratory under controlled conditions, avoiding exposure to direct sunlight and excessive heat. Prior to analysis, samples were homogenized at room temperature without applying any heat treatment or filtration in order to preserve their natural pollen content, mineral composition, and volatile characteristics. No biological replication at the apiary or seasonal level was included in the present study, as each honey sample represented an independent production unit collected from a distinct apiary. However, the expanded dataset included multiple samples belonging to the same botanical honey types, including chestnut and *Myosotis* honeys, allowing a broader assessment of compositional variability. Therefore, the results primarily reflect variability among individual honey samples and regions rather than within-apiary or seasonal reproducibility. One limitation of the study is the relatively small sample size, which reflects the exploratory nature of the research. However, the use of multiple analytical techniques and multivariate statistical methods enhances the robustness of the findings.

### 2.2. Melissopalynological Analysis

Melissopalynological analysis was carried out to determine the botanical origin and pollen composition of the honey samples according to the classical methodology proposed by [2] and later standardized and widely applied in honey characterization studies, including the procedure described by [1].

For pollen preparation, 10 g of each honey sample was homogenized and transferred into centrifuge tubes, followed by the addition of 20 mL of distilled water. The samples were incubated in a water bath at approximately 45 °C for 10–15 min to ensure complete dissolution. The suspensions were then centrifuged at 3500 rpm for 45 min, and the supernatant was carefully discarded.

The sediment obtained at the bottom of the tubes was mixed with a small amount of basic fuchsin–stained glycerin–gelatin and mounted onto microscope slides. The slides were gently heated at 30–40 °C, covered with an 18 × 18 mm coverslip, and left in an inverted position for approximately 12 h before microscopic examination.

Pollen identification was performed using a light microscope by comparing observed pollen grains with reference slides and palynological literature. For each sample, a total of 500 pollen grains were counted to ensure statistical reliability of the pollen spectrum and to support the classification of honey samples based on pollen dominance criteria. Identified pollen types were classified according to their relative frequencies as dominant (≥45%), secondary (16–44%), minor (3–15%), and rare (<3%), following the criteria established by [2]. Honey samples containing a pollen type exceeding 45% were classified as monofloral, while the remaining samples were considered multifloral [7].

### 2.3. Moisture Content

The moisture content of the honey samples was determined according to the method described by [8] and applied by [1]. Moisture analysis was carried out using a portable refractometer at room temperature.

Prior to measurement, the refractometer was calibrated with distilled water. A small amount of homogenized honey was placed on the prism surface, and moisture values were recorded directly as percentages (%). All measurements were performed in duplicate, and the mean values were used for further evaluation.

### 2.4. Proline Content

The proline content of the honey samples was determined according to the harmonised methods of [9], as applied by [1], using a spectrophotometric method based on the ninhydrin reaction.

Briefly, honey samples were prepared and reacted with ninhydrin reagent, allowing proline to form a colored complex. The absorbance of the resulting solution was measured at a wavelength of 520 nm using a spectrophotometer. Distilled water was used as the blank, and proline standard solutions were used for calibration. Proline content was calculated from the calibration curve and expressed as mg kg^−1^ of honey.

### 2.5. Aroma Analysis

The volatile aroma compounds of the honey samples were analyzed using headspace solid-phase microextraction (HS-SPME) coupled with gas chromatography–mass spectrometry (GC–MS), following the method described by [10] with minor modifications.

For HS-SPME extraction, a 1:1 (*w*/*w*) mixture of honey and 30% NaCl solution was prepared. The samples were placed in 20 mL amber vials, sealed with PTFE-silicone septa, and equilibrated at 60 °C for 15 min under continuous stirring. Volatile compounds were then extracted using an 85 µm carboxen/polydimethylsiloxane (CAR/PDMS) fibre for 30 min. GC–MS analysis was performed using a GC-MS (GCMS-QP2010 Ultra; Shimadzu Corp., Kyoto, Japan) device with a 30 m 5-Ms column (30 m × 0.25 mm × 0.25 µm). The injector temperature was set to 250 °C, and desorption was carried out for 5 min. The oven temperature program started at 40 °C (held for 4 min), increased to 90 °C at 3 °C/min, then to 130 °C at 4 °C/min (held for 4 min), and finally to 240 °C at 5 °C/min, where it was held for 8 min. Helium (purity ≥ 99.999%) was used as the carrier gas at a constant flow rate of 1.2 mL/min. The mass spectrometer was operated in electron impact mode at 70 eV, with a scan range of m/z 30–600 and a split ratio of 1:10.

Volatile compounds were identified by comparing their mass spectra with Wiley and NIST libraries, as well as by calculating retention indices using a C8–C32 n-alkane series. Quantification was performed using a relative approach based on peak area normalization (% area). No internal standard was used. Due to the lack of authentic standards for all identified compounds, results should be interpreted as approximate concentrations.

### 2.6. Element (Trace Element) Analysis

Elemental analysis of the honey samples was performed according to the method described by [11] using inductively coupled plasma–optical emission spectrometry (ICP-OES).

For sample preparation, 300 mg of each honey sample was accurately weighed into Teflon digestion vessels. A mixture of 5 mL concentrated nitric acid (HNO_3_) and 3 mL hydrogen peroxide (H_2_O_2_) was added to each vessel. The samples were digested using a microwave-assisted wet digestion system under controlled conditions. After digestion, the solutions were allowed to cool and were diluted to an appropriate volume with ultrapure water.

The concentrations of selected elements, including Cd, Cu, Fe, Mn, Na, Ni, Pb, Zn, B, Mg, Ca, Al and K were determined using an ICP-OES instrument (Optima 7000 DV) (PerkinElmer Inc., Waltham, MA, USA). The operating conditions of the ICP-OES were set as follows: RF power 1.0 kW, plasma gas flow (Ar) 15 L/min, auxiliary gas flow (Ar) 1.5 L/min, nebulizer gas flow (Ar) 0.75 L/min, RF generator frequency 40 MHz, and pump speed 15 rpm.

Calibration was performed using five multi-element standard solutions within a concentration range of 0.02–1.0 mg L^−1^, and calibration curves with correlation coefficients (R^2^ > 0.99) were accepted. Element concentrations were expressed as mg kg^−1^ of honey. Limits of detection (LOD) and limits of quantification (LOQ) were determined based on 3× and 10× the standard deviation (SD) of replicate blank measurements, respectively. The calculated LOD and LOQ values varied depending on the element and wavelength used, with, for example, LOD values of 0.0003 mg/L for Cd and 0.0288 mg/L for Pb, and corresponding LOQ values of 0.0020 mg/L for Cd and 0.0390 mg/L for Pb. Blank measurements were included in the calculation of detection limits, and all reported concentrations were above the corresponding LOQ values.

### 2.7. Statistical Analysis

Statistical analysis was performed using non-parametric methods due to the limited sample size and non-normal distribution of the data. The Kruskal–Wallis test was applied to evaluate differences among samples. Prior to multivariate analysis, data were normalized and autoscaled (mean-centered and divided by standard deviation). Principal Component Analysis (PCA) and Hierarchical Cluster Analysis (HCA) were used as exploratory tools to investigate patterns in the dataset. Each honey sample represents a single independent production unit. No biological replication was included in the study; therefore, the results reflect single-sample observations rather than replicated experimental measurements.

## 3. Results

The melissopalynological analysis revealed considerable variation in pollen composition among the ten honey samples collected from the Varda Plateau (Rize) and Yusufeli (Artvin) regions. According to the pollen frequency criteria, two samples were classified as monofloral chestnut honey, three samples as *Myosotis*-dominant honey, and the remaining samples as multifloral honeys.

*Castanea sativa* pollen was identified as the dominant pollen type in samples V4 and V10, accounting for 99% and 90% of the total pollen spectrum, respectively. These values clearly exceeded the threshold generally accepted for monofloral chestnut honey. In contrast, *Myosotis* pollen was dominant in samples V5 (56%), V7 (80%), and V8 (50%), indicating a strong contribution of Boraginaceae flora to the nectar source of these honeys.

The remaining samples (V1, V2, V3, V6, and V9) exhibited multifloral characteristics, containing pollen from various botanical taxa without a single dominant pollen type. In these samples, *Myosotis* was present as secondary pollen in V2 (25%), V3 (18%), and V9 (20%). *Rhododendron* spp. reached 20% in V6, while Eucalyptus spp. constituted 10% of the pollen spectrum in both V7 and V8. *Onobrychis* spp. was detected at 10% in V7. Several additional taxa, including Fabaceae, Brassicaceae, Rosaceae, Asteraceae, Apiaceae, Campanula, Cistus, and *Rhododendron*, occurred as minor or rare pollen types across the samples.

Fabaceae pollen was the most widely distributed taxon and was detected in all honey samples, although only at low frequencies. Similarly, Asteraceae, Brassicaceae, Rosaceae, and other herbaceous taxa contributed to the overall botanical diversity of the honey samples. The presence of these taxa reflects the rich floral resources of the Eastern Black Sea and northeastern Anatolian ecosystems.

Overall, the pollen analysis demonstrated a clear differentiation between *Castanea sativa* honeys, *Myosotis*-dominant honeys, and multifloral honeys, confirming the heterogeneous botanical origins of the investigated samples (Table 1).

Moisture content ranged from 15.8% to 21.7%, while proline values varied between 648 and 1150 mg kg^−1^. Most samples exhibited moisture values at or below 20%, suggesting adequate maturity. However, samples V3 (21.0%) and V5 (21.7%) exceeded this commonly accepted threshold, which may indicate a higher susceptibility to fermentation or reflect specific environmental or harvesting conditions.

All honey samples showed proline contents significantly above the minimum threshold of 300 mg kg^−1^. The highest proline content was recorded in sample V5 (*Myosotis* honey), whereas the lowest value was observed in sample V6 (Table 2).

A total of 114 volatile compounds belonging to eleven chemical classes were identified in the ten honey samples (V1–V10) by HS-SPME/GC–MS analysis (Supplementary Table S1). The detected compounds were classified as alcohols, aldehydes, esters and lactones, furans, ketones, organic acids, hydrocarbons, terpenes and terpenoids, sulfur compounds, nitriles, and miscellaneous compounds.

Alcohols and aldehydes represented major fractions in several samples. Isoamyl alcohol was one of the predominant alcohols, reaching 24.22% in V10 and 7.29% in V7, while phenethyl alcohol was particularly abundant in V7 (21.27%). Among aldehydes, benzaldehyde was detected in most samples and was especially abundant in V1 (15.36%), V2 (11.29%), and V8 (11.44%). Furfural was another widely distributed aldehyde, showing its highest proportion in V4 (12.90%).

Esters and lactones constituted an important fraction of the volatile profile, particularly in samples V2–V5. Hex-3(Z)-enyl butyrate was the dominant ester in V3 (25.61%), V4 (15.13%), and V5 (12.47%), whereas amyl formate was highly abundant in V2 (18.71%) and V5 (9.84%). Diethyl phthalate was detected in several samples, with the highest proportions observed in V8 (17.98%), V7 (16.62%), and V10 (13.85%).

Hydrocarbons were mainly represented by nonane, which was detected in eight samples and reached its highest relative abundance in V6 (20.30%), followed by V1 (17.68%) and V9 (14.20%). Decane was characteristic of V7, accounting for 7.69% of the total volatile composition.

Marked differences were also observed in the terpene and terpenoid fraction. Trans-linalool oxide was dominant in V3 (15.64%) and V5 (9.70%), whereas linalool reached 15.99% in V8. Farnesol (cis,cis-) was the most abundant individual volatile compound detected in the dataset, representing 59.12% of the total volatile composition in V9. In addition, V10 was characterized by relatively high proportions of p-cymen-8-ol (5.37%), pulegone (4.76%), and isoborneol (4.80%).

Sulfur-containing compounds were detected only in a limited number of samples. Butane-2-thiol (3-methyl-) was identified at relatively high levels in V3 (6.58%) and V5 (5.64%), while 3-mercapto-2-butanone was detected in V6 (4.51%). Hydroxymethylfurfural was found only in V6 and V9, accounting for 6.66% and 2.63%, respectively.

Overall, the volatile profiles revealed considerable compositional variability among the honey samples, with distinct dominance patterns of alcohols, aldehydes, esters, hydrocarbons, and terpenoid compounds depending on sample origin and botanical source (Supplementary Table S1). The interpretation of volatile compound data should be considered with caution, as the analysis was based on relative peak area percentages without the use of an internal standard. Therefore, the results reflect comparative differences in volatile profiles rather than absolute concentrations.

The elemental composition of the honey samples (V1–V10) showed notable variability across both major and trace elements. Potassium (K) was the predominant element in all samples, with concentrations ranging from 131.47 mg kg^−1^ (V8) to 2162.00 mg kg^−1^ (V4). Sodium (Na) levels varied between 80.87 and 335.23 mg kg^−1^, while calcium (Ca) exhibited a wider distribution, with the lowest value in V7 (17.51 mg kg^−1^) and the highest in V2 (1250.91 mg kg^−1^). Magnesium (Mg) concentrations ranged from 22.36 mg kg^−1^ (V8) to 93.50 mg kg^−1^ (V4).

Among trace elements, iron (Fe) concentrations ranged from 2.10 to 55.82 mg kg^−1^. The highest Fe levels were observed in samples V10 (55.82 mg kg^−1^), V8 (54.79 mg kg^−1^), V9 (52.92 mg kg^−1^), and V7 (46.21 mg kg^−1^), whereas the lowest concentration was detected in V4 (2.10 mg kg^−1^). Zinc (Zn) showed substantial variation, with markedly higher levels in V3 (141.98 mg kg^−1^) and V2 (102.14 mg kg^−1^) compared to the remaining samples. Manganese (Mn) concentrations ranged from non-detectable levels in V1 to 16.75 mg kg^−1^ in V4. Nickel (Ni) values varied between non-detectable levels and 3.91 mg kg^−1^, with the highest concentration observed in V5. Boron (B) concentrations ranged from 0.54 to 3.20 mg kg^−1^.

Copper (Cu) concentrations ranged from 0.55 to 9.11 mg kg^−1^, with the highest values recorded in V8, V9, and V7. Cadmium (Cd) was detected at low levels between 0.06 and 0.20 mg kg^−1^. Lead (Pb) was not detected in most samples, while measurable concentrations were observed in V4 (0.25 mg kg^−1^) and V5 (0.47 mg kg^−1^). Aluminum (Al) concentrations ranged from 5.11 mg kg^−1^ (V10) to 66.08 mg kg^−1^ (V8) (Supplementary Table S1).

## 4. Discussion

The present study was designed as a comparative characterization of independently produced honey samples collected from two geographically distinct regions of northeastern Türkiye. Although the number of samples within each botanical category remains limited and no biological replication at the apiary or seasonal level was included, the dataset encompasses multiple chestnut and *Myosotis* honeys as well as multifloral samples from different ecological environments. Therefore, the results provide a valuable overview of the compositional variability associated with both botanical and geographical origins. Given the exploratory nature of the study and the absence of replicated sampling, the statistical evaluation was focused on descriptive analyses and multivariate exploratory techniques (PCA, HCA, and UpSet analysis), which are widely used for pattern recognition and sample differentiation in honey characterization studies. Future investigations incorporating larger sample sets, multi-season sampling, and replicated experimental designs would further improve the statistical robustness of the observed compositional trends and facilitate a more detailed assessment of temporal and regional variability.

Samples V4 and V10 were characterized by exceptionally high proportions of *Castanea sativa* pollen (99% and 90%, respectively), allowing their unequivocal classification as monofloral chestnut honeys. The dominance of *Castanea sativa* pollen in these samples is consistent with numerous studies conducted in the Eastern Black Sea region, including Anzer, Ayder, Senoz, and other mountainous areas of northeastern Türkiye, where chestnut forests constitute one of the most important nectar sources at mid-elevation forest belts [11,12,13,14]. Similar dominance levels of *Castanea sativa* pollen have been reported for monofloral chestnut honeys from Ayder Plateau, Gümüşhane, and other Black Sea localities, confirming the strong nectar contribution of chestnut during its flowering period in the region [12,15]. The presence of two independent chestnut honey samples from different geographical locations in the present study further supports the widespread influence of *Castanea sativa* as a major nectar source in northeastern Türkiye.

Samples V5, V7, and V8 were characterized by dominant *Myosotis* pollen frequencies of 56%, 80%, and 50%, respectively, exceeding the generally accepted monofloral threshold of 45%. Accordingly, these samples were classified as monofloral *Myosotis* honeys based on pollen dominance criteria. *Myosotis* honey has previously been reported from Şırnak Province, where two unifloral *Myosotis* honey samples were identified among honeys collected from the Beytüşşebap district [16]. This finding demonstrated that *Myosotis* species can act as a dominant nectar source under suitable ecological conditions. Although *Myosotis*-dominant honeys are reported less frequently than chestnut honeys, several studies from high-altitude and transitional zones of Eastern Anatolia and the Black Sea region have identified *Myosotis* pollen as an important nectar source, particularly in subalpine meadows and open grasslands [15,17]. The occurrence of three monofloral *Myosotis* honey samples originating from two different geographical regions in the present study further confirms the significant nectar contribution of *Myosotis* species in northeastern Türkiye. These findings highlight the ecological importance of herbaceous highland flora and indicate that local environmental conditions can promote *Myosotis* species as a primary nectar source during specific flowering periods.

The remaining samples (V1, V2, V3, V6 and V9) were classified as multifloral honeys, as no single pollen taxon reached dominance. These samples exhibited a broad pollen spectrum comprising taxa from Fabaceae, Asteraceae, Brassicaceae, Rosaceae, Boraginaceae, Ericaceae, and Lamiaceae families. The frequent detection of Fabaceae pollen, including genera such as Trifolium, Onobrychis, and Astragalus, aligns with previous studies from Kars, Tunceli, Gümüşhane, and Anzer regions, where Fabaceae species are consistently reported as major nectar and pollen sources in highland ecosystems [17].

Minor and rare pollen taxa such as *Rumex*, Ranunculaceae, Apiaceae, *Plantago*, and *Cistus* were also identified across multifloral samples. The presence of these taxa is characteristic of highland multifloral honeys, where short flowering seasons, climatic variability, and heterogeneous vegetation prevent the dominance of a single plant species. Comparable pollen diversity has been reported for multifloral honeys from Anzer and Komati plateaus, supporting the view that high-altitude honeys generally exhibit complex pollen assemblages rather than strict monofloral patterns [5,6,15,18].

Overall, the melissopalynological findings demonstrated a clear differentiation of the investigated honey samples into chestnut-dominant, *Myosotis*-dominant, and multifloral honey types. These results are consistent with previous studies conducted in the Eastern Black Sea and northeastern Anatolian regions and confirm the significant contribution of both forest-dominated vegetation and herbaceous highland flora to honey production. The occurrence of multiple *Myosotis*-dominant and chestnut honeys originating from different geographical locations further highlights the ecological diversity of the study area and emphasizes the importance of regional floral resources in shaping honey botanical origin. Collectively, the pollen spectra revealed considerable floristic heterogeneity and supported the classification of the investigated honeys according to their dominant nectar sources.

Moisture content is a critical quality parameter affecting honey stability, fermentation risk, and shelf life. In the present study, most samples exhibited moisture values close to or below the commonly accepted limit of 20% by the Turkish Food Codex and European Union standards. However, samples V3 and V5 slightly exceeded this threshold, which suggests that not all samples fully meet standard moisture criteria and highlights the influence of environmental conditions such as high humidity and early harvesting. Similar moisture ranges (16.9–19.1%) were reported for honeys from Cimil Plateau (Rize) by [19], emphasizing that honeys produced in the İkizdere Valley generally comply with quality standards despite the region’s high precipitation and humidity. Most samples were within commonly accepted limits; however, some exceeded these thresholds, indicating that compliance cannot be generalized to all samples.

The relatively higher moisture contents observed in samples V3 and V5 may be associated with early harvesting or local microclimatic conditions, which are common in high-altitude and high-humidity environments of the Eastern Black Sea region (Table 2). Comparable findings were reported for Ayder and Anzer honeys, where moisture values occasionally approached the upper legal limits due to frequent rainfall and short flowering periods [1,13].

Proline content, which is widely recognized as an indicator of honey maturity, authenticity, and botanical origin, showed substantial variation among the samples. All analyzed honeys exhibited proline values well above the minimum limit of 300 mg kg^−1^, supporting their natural origin and absence of adulteration. The proline values obtained in this study are notably higher than those reported by [1] for monofloral and multifloral honeys from different regions of Türkiye (503–696 mg kg^−1^), indicating a strong contribution of both bee activity and floral sources in İkizdere Valley honeys.

The highest proline content was detected in sample V5 (monofloral *Myosotis* honey), suggesting that *Myosotis* species may contribute significantly to amino acid accumulation. This finding is particularly noteworthy, as studies on highland honeys have shown that herbaceous flora can result in elevated proline levels, comparable to or even exceeding those of well-known monofloral honeys. In contrast, V4 (monofloral chestnut honey) exhibited moderately high proline content, which aligns with previous studies reporting variable proline levels in chestnut honeys depending on region and nectar flow intensity.

Similar proline ranges (568–758 mg kg^−1^) were reported for Cimil Plateau honeys by [19], while higher proline values were observed in some Anzer honeys (808–1139 mg kg^−1^), likely due to the exceptionally rich and endemic flora of that region. The proline values obtained in the present study fall within or above the ranges reported for other highland honeys from Rize and surrounding provinces, confirming the high biochemical quality of İkizdere Valley honeys.

Overall, the combined evaluation of moisture and proline contents indicates that the honey samples analyzed in this study are generally well matured and exhibit characteristics consistent with natural honey quality, despite the diverse environmental conditions of the investigated regions. The observed variations further highlight the influence of botanical origin, particularly in monofloral chestnut honeys (V4 and V10) and *Myosotis* honeys (V5, V7, and V8), on key physicochemical parameters. Although proline content and pollen composition are widely used as indicators of honey maturity and botanical origin, they should not be considered definitive proof of authenticity or the absence of adulteration. Rather, these parameters provide supportive evidence that should be complemented by additional analytical approaches. All analyzed honey samples exhibited proline concentrations substantially above the commonly accepted minimum threshold for natural honey, supporting their adequate maturation and natural origin. Nevertheless, proline content alone cannot be regarded as a conclusive indicator of authenticity, emphasizing the importance of a multidisciplinary approach for honey characterization and quality assessment.

In V1, aldehydes were the predominant class, with benzaldehyde (15.36%) and pelargonaldehyde (11.50%) as the most abundant compounds. Furfural (6.39%) and phenylacetaldehyde (4.48%) also contributed significantly to the profile. Among hydrocarbons, nonane (17.68%) represented the highest single compound, while alcohols such as isoamyl alcohol (4.91%) and cis-linalool oxide (5.96%) were present at moderate levels.

The V2 sample was characterized by a high proportion of esters and aldehydes. Amyl formate (18.71%) was the dominant compound, followed by benzaldehyde (11.29%) and phenylacetaldehyde (9.37%). Alcohols such as trans-linalool oxide (5.61%) and isoamyl alcohol (2.06%) were also detected. In addition, organic acids, particularly butyric acid (4.17%), were present, indicating a more complex volatile composition.

In V3, esters and alcohols clearly dominated the volatile profile. Hex-3(Z)-enyl butyrate (25.61%) was the most abundant compound, accompanied by a high level of trans-linalool oxide (15.64%). Alcohols such as isoamyl alcohol (5.30%) were also prominent. Aldehydes, including furfural (2.62%) and benzaldehyde (1.80%), were detected at comparatively lower levels. Linalool and its oxide derivatives are well-recognized floral markers and have been frequently reported in honeys with pronounced fresh and floral characteristics [20].

The V4 sample exhibited a distinct volatile composition, with aldehydes, esters, and hydrocarbons contributing substantially to the overall profile. Furfural (12.90%) and hex-3(Z)-enyl butyrate (15.13%) were the main compounds. Hydrocarbons such as dimethylstyrene (11.43%) and p-cymene (6.11%) were also notable. Among alcohols, verbenol (8.96%) and isoborneol (3.59%) were present at relatively high levels. The detection of p-cymene, verbenol, and isoborneol suggests the contribution of terpene-related compounds, which are commonly linked to botanical origin and resinous or herbal notes [21].

**Figure 2:**
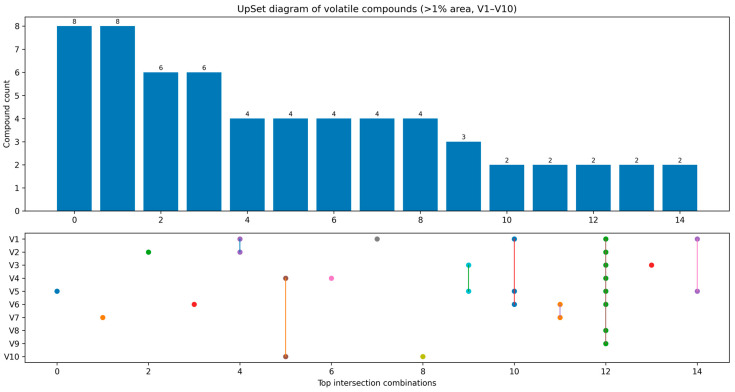
UpSet diagram illustrating shared and volatile compounds among V1–V10 samples.

In V5, esters constituted the dominant chemical class. The most abundant compounds were hex-3(Z)-enyl butyrate (12.47%), amyl formate (9.84%), and isoamyl butyrate (9.77%). Alcohols, including trans-linalool oxide (9.70%) and isoamyl alcohol (3.09%), also contributed significantly, while aldehydes were detected at lower relative percentages. Ester-rich profiles have been reported in honeys characterized by intense fruity and green sensory attributes, emphasizing the role of esters in differentiating honey aroma profiles [22,23]. In the UpSet diagram, V5 exhibited the highest number of sample-specific volatile compounds among the *Myosotis* honeys. The bars corresponding to the V5-only set indicate several unique compounds, including hept-3(Z)-en-1-ol, dihydromyrcenol, ethyl nonanoate, isoamyl isobutyrate, and undeca-1,3,5-triene, which were not observed in any other honey samples. Sample V5 (*Myosotis*-dominant) exhibited a volatile profile that differed from the other analyzed samples, suggesting potential compositional particularities associated with this botanical source. However, as this observation is based on a single sample, it should be interpreted with caution and cannot be considered representative of *Myosotis* honey in general (Figure 2).

Sample V6 differed markedly from the other samples due to its elevated ketone content. Pyruvophenone (14.68%), heptylidene acetone (4.76%), and isophorone (4.50%) were the dominant ketones detected in this sample. Ketone-rich volatile profiles are relatively uncommon in honey; however, compounds such as isophorone and related ketones have previously been associated with specific botanical sources and oxidative transformations [21]. Aldehydes, including hydroxymethylfurfural (6.66%) and benzaldehyde (3.99%), were also present at appreciable levels. The occurrence of hydroxymethylfurfural together with benzaldehyde may reflect additional processing- or storage-related influences, as HMF is widely recognized as an indicator of honey aging and thermal exposure [11]. Similar to V1, nonane (20.30%) was the most abundant hydrocarbon in V6.

Recent large-scale studies employing HS-SPME-GC-MS in combination with chemometric approaches have demonstrated high classification accuracy for both botanical and geographical origins of honey [24]. Compared with these extensive datasets, the present study should be regarded as an exploratory investigation; therefore, the proposed interpretations are intended to be indicative rather than definitive.

Despite the pronounced variability among samples, several volatile compounds were widely distributed throughout the dataset. Benzaldehyde, furfural, caprylic aldehyde, and nonane were detected in numerous samples, although their relative abundances varied considerably (Figure 2). Likewise, linalool and its oxide derivatives (cis- and trans-linalool oxide) were identified in most samples, contributing floral and fresh aromatic characteristics to the honey aroma profiles. The recurrent occurrence of benzaldehyde, furfural, and nonane agrees with previous reports on Turkish honeys [14], where these compounds were also recognized as common volatile constituents. These compounds are known to contribute almond-like, caramelized, and background aromatic notes to honey. The frequent detection of linalool derivatives in the present study suggests a strong floral influence, likely associated with differences in botanical origin among the investigated samples.

The monofloral chestnut honeys (V4 and V10) and *Myosotis* honeys (V5, V7, and V8) exhibited more distinctive volatile profiles than the multifloral samples. Sample V4 was characterized by relatively high levels of furfural, verbenol, and aromatic hydrocarbons, whereas V10 showed elevated proportions of isoamyl alcohol, isoborneol, p-cymen-8-ol, and pulegone. In contrast, the *Myosotis* honeys displayed different dominant volatile constituents, including ester-rich profiles in V5, high phenethyl alcohol content in V7, and elevated linalool levels in V8. These findings indicate that even within the same botanical honey group, volatile composition may vary according to geographical and environmental conditions.

The UpSet analysis revealed a complex pattern of shared and unique volatile compounds among the honey samples. While several compounds were common to multiple samples, numerous volatiles were restricted to specific honey types or geographical origins. The monofloral chestnut and *Myosotis* honeys displayed comparatively lower overlap with the multifloral samples and contained characteristic volatile compounds that contributed to their distinct chemical fingerprints. Furthermore, the differentiation observed between the Varda (V1–V6) and Yusufeli (V7–V10) samples supports the combined influence of botanical and geographical origin on honey volatile composition (Figure 2).

The volatile profiles of the chestnut honey samples (V4 and V10) showed both agreement and divergence with literature data. In line with previous studies, the occurrence of furfural and terpene-related compounds such as p-cymene, verbenol, isoborneol, and p-cymen-8-ol supports the characteristic woody, resinous, and aromatic features commonly associated with chestnut honey [11]. These compounds have frequently been reported in monofloral chestnut honeys and are considered useful indicators of botanical origin. Sample V4 was characterized by relatively high proportions of furfural, verbenol, and p-cymene, whereas V10 exhibited elevated levels of isoamyl alcohol, isoborneol, p-cymen-8-ol, and pulegone. Although both samples were classified as monofloral chestnut honeys based on their dominant *Castanea sativa* pollen content, the observed differences in volatile composition suggest that geographical origin and local environmental conditions may substantially influence the aromatic profile of chestnut honey. Similar variability among chestnut honeys from different regions has been reported previously and reflects the combined effects of floral source, climate, and surrounding vegetation on honey aroma composition.

However, a notable difference is the absence of aminoacetophenone, which has been proposed in the literature as a key marker compound for chestnut honey [14]. Its absence in the present sample suggests that the volatile fingerprint of chestnut honey may vary depending on geographical origin, environmental conditions, or analytical methodology. In addition, the relatively high abundance of hex-3(Z)-enyl butyrate, an ester typically associated with green and fruity notes, represents a divergence from previously reported chestnut honey profiles, where aldehydes and benzene derivatives are usually more dominant.

Overall, although the chestnut honey samples (V4 and V10) shared several characteristic aromatic compounds previously reported for monofloral chestnut honeys, the differences observed between their volatile profiles highlight the natural variability of this honey type. These findings suggest that the aroma profile of chestnut honey is shaped by a combination of botanical, geographical, and environmental factors rather than by a single universal marker compound. Consequently, the authentication and characterization of chestnut honey should rely on the overall volatile fingerprint rather than on the presence of individual compounds alone.

In contrast, the multifloral samples (V1, V2, V3, V6 and V9) participate in a greater number of intersections and share a larger pool of volatile compounds, resulting in more complex and overlapping profiles. Overall, the UpSet diagram clearly suggests that monofloral honeys exhibit more selective and distinctive volatile compositions, whereas multifloral honeys show broader compound overlap, supporting the role of botanical origin in shaping honey aroma profiles.

The multifloral samples exhibited greater variability in their volatile compositions than the monofloral honeys. Samples V2 and V6, which contained several secondary pollen sources, displayed particularly heterogeneous volatile profiles. In V2, the coexistence of esters, aldehydes, alcohols, and organic acids contributed to a diversified aroma composition. In contrast, V6 was distinguished by its elevated ketone content, primarily represented by pyruvophenone, heptylidene acetone, and isophorone, resulting in a more pronounced and complex volatile pattern. Sample V9 also exhibited a distinctive profile among the multifloral honeys due to the predominance of farnesol, highlighting the substantial influence of specific volatile constituents on the overall aroma composition. These findings indicate that multifloral honeys may encompass a broader range of volatile compounds reflecting the contribution of multiple nectar sources.

The observed variations in elemental composition among samples V1–V10 likely reflect differences in soil–plant interactions, floral sources, and environmental conditions rather than purely geochemical processes. In honey, elemental composition is primarily influenced by soil–plant transfer mechanisms, floral source, altitude, and local environmental conditions affecting nectar composition.

The elevated potassium levels observed in the chestnut honey sample V4 and the higher calcium concentrations detected in sample V2 may be associated with differences in plant species and their nutrient uptake patterns from the soil. Likewise, the remarkably high iron concentrations recorded in the Yusufeli samples (V7–V10), together with the elevated zinc levels observed in V2 and V3, indicate that elemental composition is strongly influenced by both botanical and geographical factors. Variations in trace elements such as Zn, Mn, Fe, and Cu may further reflect localized environmental conditions, including soil characteristics, geological background, and microclimatic factors. These findings are consistent with previous studies reporting that the mineral composition of honey is shaped by a complex interaction between floral source and regional environmental conditions, making elemental profiles useful complementary indicators for geographical and botanical discrimination.

The relatively consistent distribution of Fe across samples suggests a common background contribution, while the presence of elements such as Ni, Pb, and Cd in certain samples may indicate localized environmental inputs, including possible anthropogenic sources. In this context, the elemental composition of honey should be interpreted as the result of complex biological and environmental interactions rather than purely geochemical processes.

Unlike the relatively homogeneous Fe concentrations observed in the Varda samples (V1–V6), the inclusion of the Yusufeli samples (V7–V10) revealed substantially higher iron levels, indicating a strong geographical influence on elemental composition. The pronounced enrichment of Zn in samples V2 and V3 suggests localized accumulation or preferential incorporation associated with specific floral and environmental conditions. Similarly, the elevated Mn concentration observed in the chestnut honey sample V4 may reflect mineral-specific enrichment and the influence of botanical origin. The distinct distribution patterns of Fe, Zn, and Mn across the investigated samples further support the usefulness of elemental composition as a complementary tool for differentiating honey according to both geographical and botanical origins.

Trace elements generally occurred at low concentrations; however, their heterogeneous distribution provides valuable insights into localized environmental and geochemical influences. The elevated concentrations of nickel (Ni) and boron (B) observed in sample V5 suggest a potential association or co-enrichment of these elements under specific environmental conditions. In contrast, Ni was absent or detected only at very low levels in most of the remaining samples, highlighting considerable spatial variability in trace element distribution. The occurrence of lead (Pb) was restricted to samples V4 and V5, whereas it was not detected in the other honey samples. This localized distribution pattern suggests site-specific enrichment rather than a uniform regional source and may be associated with differences in geological background, soil composition, or environmental inputs. Overall, the heterogeneous distribution of trace elements supports the influence of both botanical and geographical factors on the elemental composition of honey.

Although Pb was not detected in most samples, measurable concentrations were observed in V4 (0.25 mg/kg) and V5 (0.47 mg/kg). These values are noteworthy because they exceed the maximum level of 0.10 mg/kg established for honey under Commission Regulation (EU) 2023/915. Therefore, the presence of Pb in these samples should be considered not only as an indicator of elemental variability but also as a potential food safety concern. Possible sources of Pb contamination in honey may include atmospheric deposition, soil-derived uptake by plants, proximity to roads or anthropogenic activities, beekeeping equipment, processing materials, and storage containers. Since the present study was not designed as a formal dietary exposure or risk assessment, no definitive conclusion can be drawn regarding consumer health risk. Nevertheless, the elevated Pb levels detected in V4 and V5 indicate the need for further targeted monitoring, repeated sampling, and source-identification studies in order to determine whether these findings reflect localized environmental contamination or sample-specific contamination during handling or storage.

**Figure 3:**
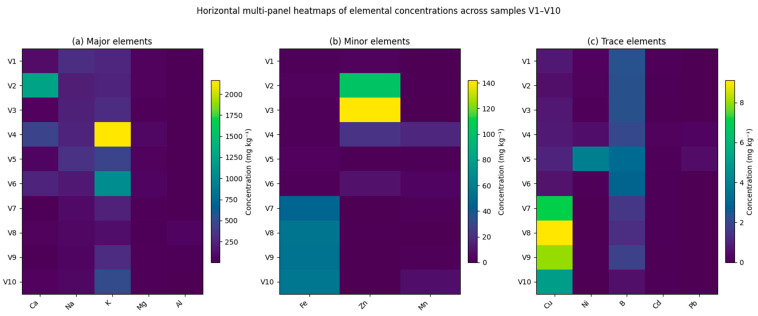
Horizontal multi-panel heatmaps illustrating the distribution of (**a**) major, (**b**) minor, and (**c**) trace elements across samples V1–V10. Each panel is independently scaled with its own colorbar to highlight compositional variability within each element group.

Overall, the heatmap-based visualization effectively highlighted the compositional heterogeneity of the investigated honey samples and revealed element associations that were not readily apparent from tabulated data alone. The distinct enrichment patterns observed for potassium in the chestnut honey sample V4, zinc in samples V2 and V3, iron in the Yusufeli samples (V7–V10), and nickel in sample V5 emphasize the considerable variability in elemental composition across the dataset. These distribution patterns support the interpretation that both botanical origin and geographical factors contribute to elemental variability (Figure 3). Furthermore, the heatmap results were consistent with the differentiation observed in the multivariate analyses, providing additional evidence that elemental composition can serve as a useful complementary tool for the characterization and discrimination of honey samples according to their botanical and regional origins.

Table 3 presents a comparative overview of the minimum and maximum concentrations of selected elements determined in the present study and those reported in previous studies conducted in different geographical regions. The comparison clearly indicates that elemental composition in honey exhibits considerable variability, which can largely be attributed to differences in geographical origin, soil composition, botanical sources, and environmental conditions.

In the present study, the concentrations of major elements such as K, Ca, Na, and Mg were within or comparable to the ranges reported in the literature. Potassium was the dominant element, showing a wide concentration range (216.64–2162.00 mg/kg), which is consistent with previous reports identifying K as the most abundant mineral in honey. Comparable K ranges have been reported for honeys from Italy, Malaysia, Iraq, and Brazil, although some studies, particularly those conducted in tropical regions, reported higher maximum values. This variability supports the strong influence of soil mineral content and plant species on potassium accumulation in honey.

Calcium and sodium concentrations in the present study were generally higher than those reported in some European studies but remained comparable to values reported from Middle Eastern and South American regions. These findings suggest that regional environmental and botanical characteristics and climatic conditions may play a significant role in shaping the mineral profile of honey. Magnesium levels also showed substantial variability across studies, with the present results falling within the broad ranges reported in global reviews.

Trace elements such as Fe, Zn, Cu, Mn, and Ni exhibited marked differences among studies. Iron concentrations in the present study were relatively narrow and lower than the extreme values reported in global datasets, indicating limited environmental contamination in the sampling area. Zinc concentrations, however, showed a wide range, overlapping with values reported in both European and Asian studies, which may reflect differences in floral origin and soil composition. Copper and manganese concentrations were generally comparable to those reported in previous studies, although higher Mn values observed in some samples may indicate localized environmental or botanical effects.

Cadmium (Cd) and lead (Pb) were detected at relatively low to moderate levels compared to values reported in the literature. However, measurable Pb concentrations in samples V4 (0.25 mg/kg) and V5 (0.47 mg/kg) should be interpreted with caution, as they may indicate localized environmental inputs. Therefore, while the observed values fall within ranges reported in previous studies, they do not necessarily imply the absence of environmental contamination. The relatively low levels observed in this study, when compared to extreme values reported in some regions, suggest a limited degree of contamination; however, the presence of detectable Pb in certain samples indicates that environmental influences cannot be entirely excluded.

Recent research has suggested that honey can act as a bioindicator of environmental contamination, including both trace metals and emerging pollutants such as microplastics [30,31,32]. In this context, the elevated Pb levels observed in some samples in the present study may reflect localized environmental pollution and reinforce the importance of monitoring trace element composition in honey.

It should also be noted that the robustness of mineral and physicochemical classification models is strongly influenced by sample size and botanical diversity, as demonstrated in recent large-scale studies involving extensive honey datasets [28]. Therefore, the findings of the present study should be interpreted as preliminary and exploratory.

Overall, the comparison demonstrates that the elemental composition of honey is highly dependent on geographical origin. The results of the present study are largely consistent with previously published data and fall within internationally reported concentration ranges. Differences observed among studies further emphasize the importance of regional environmental factors, soil geochemistry, and botanical sources in determining the mineral and trace element composition of honey.

**Figure 4:**
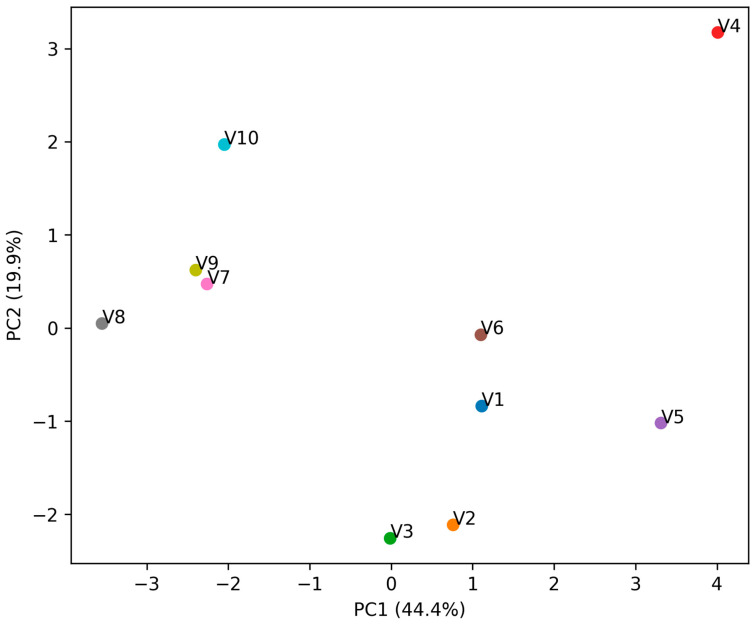
Principal Component Analysis (PCA) score plot showing the distribution of honey samples (V1–V10) based on their elemental composition.

Principal Component Analysis (PCA) was applied to explore patterns in the elemental composition of the honey samples (Figure 4). The first two principal components explained 64.3% of the total variance, with PC1 and PC2 accounting for 44.4% and 19.9%, respectively. The score plot revealed a clear separation of the Yusufeli samples (V7–V10) from the Varda samples (V1–V6), indicating a strong influence of geographical origin on elemental composition. The Yusufeli samples were primarily associated with elevated Fe and Cu concentrations, whereas the Varda samples were characterized by comparatively higher concentrations of Zn, Ca, K, and other macroelements. In addition, the monofloral chestnut honeys (V4 and V10) and *Myosotis* honeys (V5, V7, and V8) occupied distinctive positions within the PCA space, reflecting the contribution of botanical origin to elemental variability. Overall, the PCA results demonstrated considerable compositional heterogeneity among the investigated honey samples and highlighted the combined effects of geographical and botanical factors on elemental composition (Figure 4).

**Figure 5:**
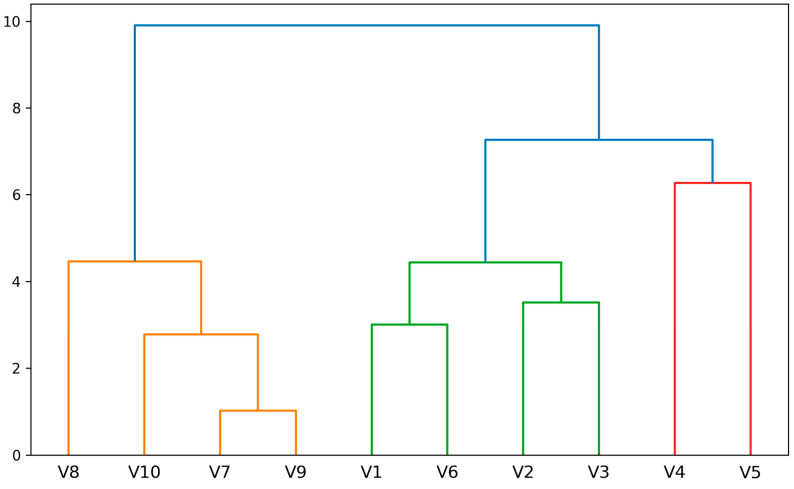
Hierarchical cluster analysis (HCA) dendrogram of honey samples (V1–V10) based on elemental composition using Ward’s method and Euclidean distance.

Hierarchical cluster analysis (HCA) revealed a clustering pattern largely consistent with the PCA results (Figure 5). The Yusufeli samples (V7–V10) formed a distinct cluster characterized by elevated Fe and Cu concentrations, whereas the Varda samples (V1–V6) were grouped separately. Within the Varda cluster, additional subclusters were observed, reflecting differences in elemental composition among the samples. In particular, V4 formed a distinct branch owing to its exceptionally high K and Mn contents, while V5 exhibited a unique clustering pattern associated with elevated Ni and B concentrations. Samples V2 and V3 clustered closely together, primarily due to their high Zn levels.

The chemometric analyses provided valuable insight into the compositional variability of the investigated honey samples. Both PCA and HCA indicated that geographical origin exerted a strong influence on elemental composition, as evidenced by the clear differentiation between the Varda and Yusufeli samples. In addition, the distinctive positions of the chestnut honeys (V4 and V10) and the *Myosotis* honeys (V5, V7, and V8) suggest that botanical origin also contributes to elemental variability. Although the present dataset is larger than that used in the initial study and includes multiple representatives of both chestnut and *Myosotis* honeys, the number of samples within each botanical category remains limited. Therefore, the observed clustering patterns should be considered indicative rather than definitive. Further studies involving larger and more geographically diverse sample sets would be valuable for confirming the robustness of these compositional trends.

**Figure 6:**
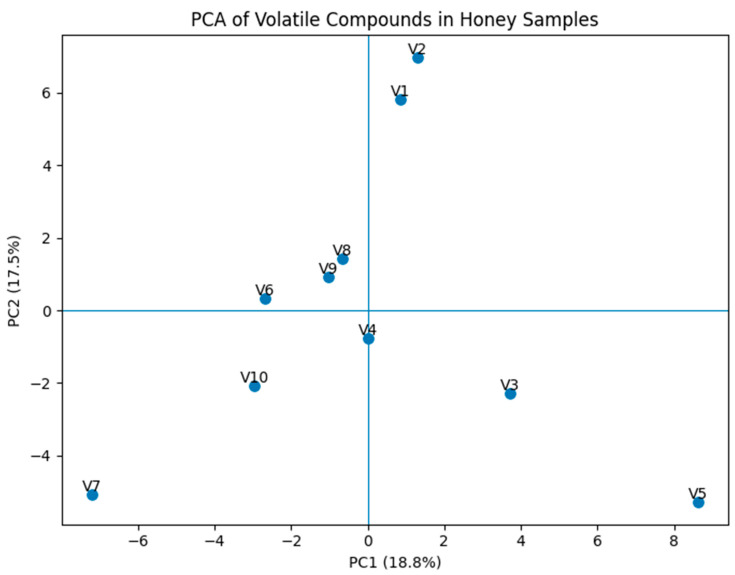
Combined Principal Component Analysis (PCA) score plot of honey samples (V1–V10) based on volatile compounds.

Principal Component Analysis (PCA) was applied to evaluate differences in volatile compound profiles among the honey samples based on compounds present at relative abundances greater than 1%. The first two principal components explained 36.3% of the total variance, with PC1 and PC2 accounting for 18.8% and 17.5%, respectively (Figure 6). The PCA score plot revealed substantial variability in volatile composition among the investigated samples.

Samples V5 and V7 were the most clearly differentiated samples within the PCA space, occupying opposite sides of the score plot and indicating markedly distinct volatile profiles. In contrast, samples V1 and V2 clustered closely together and were associated with similar aroma compositions. Samples V8 and V9 also occupied neighboring positions, suggesting a degree of similarity in their volatile fingerprints, whereas V6 and V10 were located in an intermediate region of the plot. The chestnut honey sample V4 was positioned near the center of the PCA space, indicating partial overlap with other samples despite its characteristic volatile constituents.

Overall, the PCA results demonstrated considerable heterogeneity in volatile composition and confirmed that both botanical and geographical origins contribute to the differentiation of honey aroma profiles. The observed separation patterns further support the usefulness of volatile compounds as complementary markers for honey characterization and discrimination (Figure 6).

The PCA results demonstrate that volatile compound composition is influenced by both botanical and geographical origins. Several monofloral honeys exhibited distinctive volatile fingerprints, particularly samples V5 and V7, which occupied clearly separated positions within the PCA space. In contrast, multifloral samples generally showed greater overlap, reflecting the contribution of multiple nectar sources to their aroma composition. Although the chestnut (V4 and V10) and *Myosotis* (V5, V7, and V8) honeys did not form completely discrete groups, their distribution patterns indicate that specific floral sources contribute characteristic volatile constituents that influence the overall aroma profile. These findings support the potential use of volatile compounds as complementary markers for the differentiation and characterization of honey according to both botanical and geographical origins.

A combined Principal Component Analysis (PCA) was performed using elemental composition together with physicochemical parameters (moisture and proline) to evaluate the overall compositional variability of the honey samples. The PCA score plot revealed substantial differentiation among the investigated samples, indicating that the combined dataset effectively captured both botanical and geographical variability. Samples originating from Yusufeli (V7–V10) tended to separate from the Varda samples (V1–V6), primarily due to differences in elemental composition, particularly Fe and Cu concentrations. Within the Varda group, additional differentiation was observed among samples with distinct mineral and physicochemical characteristics.

The chestnut honeys (V4 and V10) occupied characteristic positions within the PCA space, reflecting their elevated potassium, manganese, and proline contents. Likewise, the *Myosotis* honeys (V5, V7, and V8) exhibited distinctive compositional patterns compared with the multifloral samples. In contrast, several multifloral samples showed greater overlap, indicating more similar overall chemical compositions. The clustering pattern suggests that both elemental composition and physicochemical properties contribute substantially to sample discrimination.

**Figure 7:**
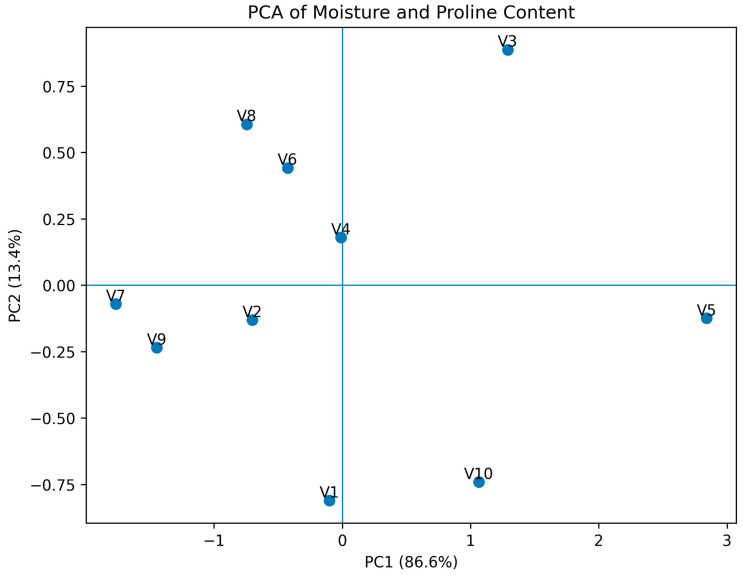
Combined Principal Component Analysis (PCA) score plot of honey samples (V1–V10) based on mineral composition and physicochemical parameters (moisture and proline).

Overall, the combined chemometric evaluation demonstrates that mineral composition and physicochemical parameters provide complementary information for honey characterization. The observed separation patterns support the influence of both botanical and geographical origins on honey composition and highlight the usefulness of integrated analytical approaches and multivariate statistics for honey authentication, classification, and origin assessment (Figure 7).

The compositional variability observed in this study likely reflects complex interactions among botanical origin, geographical location, environmental conditions, and altitude-related factors. The investigated honey samples were collected from two distinct regions, namely the Varda Plateau of the İkizdere Valley and the Yusufeli area, both characterized by diverse floristic compositions and heterogeneous environmental conditions. Variations in plant diversity, climatic conditions, geological background, and soil composition may influence nectar chemistry and mineral uptake by plants, which are subsequently reflected in the physicochemical, elemental, and volatile characteristics of honey.

The differentiation observed in the melissopalynological, elemental, physicochemical, and volatile datasets suggests that honey composition is shaped by multiple interacting factors rather than by botanical origin alone. This multifactorial influence highlights the importance of integrated analytical approaches combining conventional characterization methods with multivariate statistical analyses. At the same time, the results emphasize the need for larger and more geographically diverse datasets to further elucidate the relative contributions of botanical, environmental, and regional factors to honey composition and authenticity.

## 5. Conclusions

This study provides a comprehensive characterization of honey samples collected from the Varda Plateau (İkizdere Valley, Rize) and Yusufeli regions of northeastern Türkiye through the combined evaluation of melissopalynological, physicochemical, elemental, and volatile properties. The results demonstrated considerable compositional variability among the investigated samples and highlighted the influence of both botanical and geographical origins on honey characteristics.

Melissopalynological analysis enabled the classification of the samples into chestnut-dominant, *Myosotis*-dominant, and multifloral honey types, reflecting the rich floristic diversity of the study area. In particular, the identification of multiple *Myosotis*-dominant honeys confirms the importance of *Myosotis* species as a nectar source under suitable ecological conditions and expands current knowledge regarding this relatively uncommon honey type.

Physicochemical analyses indicated that all investigated honeys exhibited characteristics consistent with natural and well-matured honeys. Elemental composition varied substantially among samples, with potassium, calcium, iron, zinc, and manganese contributing significantly to sample differentiation. Likewise, volatile compound analysis revealed distinct aroma profiles associated with both floral source and geographical origin, while chemometric analyses (PCA, HCA, and UpSet analysis) further supported the differentiation of honey samples according to their compositional characteristics.

The combined analytical approach demonstrated that melissopalynological, physicochemical, elemental, and volatile data provide complementary information for honey characterization and origin assessment. Although the investigated sample set represents only a portion of the regional honey diversity, the observed patterns indicate that integrated analytical strategies can contribute substantially to the discrimination of honey types and the identification of compositional markers associated with botanical and geographical origins.

Overall, the present study provides new insights into the chemical and botanical characteristics of honeys from the Eastern Black Sea region and contributes to the growing body of knowledge on chestnut, *Myosotis,* and multifloral honeys. Future studies involving larger sample numbers, multi-season sampling, and advanced molecular approaches such as DNA metabarcoding will further improve the understanding, authentication, and traceability of regional honey types.

## Figures and Tables

**Table 1:** Melissopalynological analysis results of honey samples.

	V1	V2	V3	V4	V5	V6	V7	V8	V9	V10
*Castanea sativa*				99						90
Boraginaceae					22					
*Myosotis* spp.		25	18		56		80	50	20	
*Sinapis* spp.					*					
*Salix* spp.										
*Rumex* spp.		*							*	
*Trifolium* spp.		*					*			
*Sanguisorba* spp.										
Ranunculaceae						*				
Cistaceae					*				*	
Rhamnaceae										
*Papaver* spp.										
*Pinus* spp.										
*Rumex* spp.										
Asteraceae	*	*	*		*					*
Apiaceae			*		*					
*Echium* spp.									*	
*Allium* spp.										
*Plantago* spp.										
*Campanula* spp.			*							
Rosaceae		*				*				*
Poaceae										
Lamiaceae	*		*							
Fabaceae	*	*	*			*	*	*	*	*
*Onobrychis* spp.	*		*		*			10		
*Cistus* sp.	*		*							
Acanthaceae										
Brassicaceae		*			*	*		*		
*Rhododendron* spp.					*	20				*
*Paliurus* spp.									*	
*Eucaliptus* spp.								10	10	
*Geranium* spp.									*	

* indicates presence of the pollen type at a frequency below 3% (rare pollen category).

**Table 2:** Moisture and proline content of honey samples.

	V1	V2	V3	V4	V5	V6	V7	V8	V9	V10
Moisture (%)	17	17.1	21	18.4	21.7	18.2	15.8	18	16	18.6
Proline (mg kg^−1^)	907	769	874	810	1150	737	648	685	700	1025

**Table 3:** Comparative Min–Max Concentrations of Elements Reported in This Study and Reference Studies (mg/kg).

Element/Study	This Study	[25]	[26]	[27]	[28]	[29]
Region	Turkey	Italy (Europe)	Global (Multi-Continental Review)	Malaysia (Southeast Asia)	Iraq (Middle East)	Brazil (South America)
Fe	2.10–55.82	0.97–13.7	0.41–224.00	6.57–24.88	0.117–0.440	0.8–26.3
Zn	0.04–141.98	0.72–3.66	0.23–73.6	nd-4.566	-	0.142–4.276
Cu	0.55–9.11	172–5900 µg/kg	0.05–17.3	0.621–2.931	<0.10	nd-1.36
Mn	nd-16.75	0.13–16.9	0.00–4.35	-	0.0091–0.086	0.01–6.94
Ni	0.04–3.91	77–2760 µg/kg	nd-9.00	-	0.117–0.440	-
Cd	0.06–0.20	0.17–373 µg/kg	0.17–372 µg/kg	0.001–0.006	0.108–0.820	-
Pb	nd-0.47	28.2–304 µg/kg	0.63–3232.00 µg/kg	0.231–0.091	0.100–0.730	-
Ca	22.30–1250.91	137–409	4.85–218.00	309.3–76.4	140.8–240.8	28.9–262.49
Na	80.87–335.23	56–232	3.23–236.80	375.2–944.5	51.0–120.8	20.8–173.4
Mg	22.36–93.50	22.2–159	2.18–563.72	13.72–71.04	0.260–0.721	23.6–268.5
K	131.47–2162.00	147–4136	39.66–1349.34	1643.9–95.4	93.80–712.8	205.00–2639.00
Al	5.11–22.55	-	1.39–11.36	–	–	0.09–42.81
B	0.54–3.20	-	4.42–6.30	–	–	-

nd: not detected.

## Data Availability

The original contributions presented in this study are included in the article/Supplementary Materials. Further inquiries can be directed to the corresponding author.

## Supplementary Materials

The following supporting information can be downloaded at: https://www.mdpi.com/article/10.3390/foods15152593/s1, Supplementary Table S1. Relative peak area percentages (% Area) of aromatic compounds classified by chemical classes in V1–V10 samples; Supplementary Table S2. Elemental concentrations of samples V1–V10.
