# Supplementary material for: Melissopalynological, Physicochemical, Mineral, and Volatile Characterization of Castanea sativa and Myosotis Honeys in Comparison with Multifloral Honeys
Source: Foods. 2026 Jul 24;15(15):2593. doi: 10.3390/foods15152593 (PMC13464738; doi:10.3390/foods15152593)
Supplement: Supplementary file 1 [file foods-15-02593-s001.zip › foods-4391066-supplementary.pdf]

Table S1. Relative peak area percentages (% Area) of aromatic compounds classified by chemical classes in V1–V6 samples

Compound Name	Chemical Class	V1	V2	V3	V4	V5	V6	V7	V8	V9	V10
Alcohols											
Isobutyl alcohol	Alcohols	-	3.26	-	-	-	-				
Butenol <3-methyl->	Alcohols	1.27	2.71	-	-	-	-				
Isoamyl alcohol	Alcohols	4.91	2.06	5.3	-	3.09	3.2				
Phenethyl alcohol	Alcohols	-	-	1.45	-	-	-				
Prenol	Alcohols	-	1.51	-	-	-	-				
Capryl alcohol	Alcohols	1.45	-	-	-	1.73	1.15				
Butane-2-thiol <3-methyl->	Alcohols	-	-	6.58	-	5.64	-				
Hept-3(Z)-en-1-ol	Alcohols	-	-	-	-	2.01	-				
Sulcatol	Alcohols	0.39	-	-	-	-	-				
Phenol <4-ethyl->	Alcohols	-	-	-	-	-	1.43				
Hexanol <n->	Alcohols	-	-	0.59	-	-	-				
Amyl methyl carbinol	Alcohols	-	-	0.98	-	0.82	-				
Hepten-3-ol	Alcohols	-	-	-	-	0.71	-				
Vinyl amyl carbinol	Alcohols	0.81	-	-	-	-	-				
Oct-5(Z)-enol	Alcohols	-	-	0.58	-	1.38	-				
Dec-9-en-1-ol	Alcohols	-	-	-	0.99	-	-				
Verbenol	Alcohols	-	-	-	8.96	-	0.55				
Hexanol <2-ethyl->	Alcohols	2.29	1.09	0.73	1.77	1.44	1.07				
Linalool oxide <trans->	Alcohols	0.49	5.61	15.64	5.71	9.7	-				
Linalool oxide <cis->	Alcohols	5.96	1.73	4.56	2.54	-	1.07				
Dihydromyrcenol	Alcohols	-	-	-	-	0.97	-				
Linalool	Alcohols	1.94	1.24	0.73	0.46	0.96	1.78				
Isopinocampheol	Alcohols	-	-	-	-	-	0.77				
Oct-3(Z)-enol	Alcohols	-	-	-	1.82	1.32	-				
Isoborneol	Alcohols	-	-	-	3.59	-	-				
Pelargol	Alcohols	-	-	-	0.32	-	-				
Terpinen-4-ol	Alcohols	-	-	-	1.75	-	-				
Terpineol <alpha->	Alcohols	0.93	0.68	-	-	-	-				
Terpinolene	Alcohols	-	-	-	1.35	-	-				
Aldehydes											
Enanthaldehyde	Aldehydes	0.58	0.72	-	-	-	-				
Butyraldehyde <2-methyl->	Aldehydes	-	1.78	4.58	-	1.44	-				
Capronaldehyde	Aldehydes	-	-	-	-	-	2.43				

Tiglic aldehyde	Aldehydes	2.65	3.02	-	-	-	-				
Furfural	Aldehydes	6.39	5.03	2.62	12.9	1.92	4.23				
Myrtenal	Aldehydes	-	-	-	-	-	1.31				
Benzaldehyde <2.4-dimethyl->	Aldehydes	-	-	-	-	-	3.18				
Benzaldehyde	Aldehydes	15.36	11.29	1.8	0.99	0.87	3.99				
Nona-2(E).4(E)-dienal	Aldehydes	1.24	-	-	-	1.74	-				
Caprylaldehyde	Aldehydes	1.87	0.74	-	0.85	1.69	1.18				
Phenylacetaldehyde	Aldehydes	4.48	9.37	2.03	-	1.29	-				
Hendecanal	Aldehydes	2.57	-	-	-	-	-				
Pelargonaldehyde	Aldehydes	11.5	-	-	-	-	2.43				
Perillaldehyde	Aldehydes	-	-	-	0.49	-	-				
Safranal	Aldehydes	-	-	1.63	-	0.74	-				
Capraldehyde	Aldehydes	0.91	-	-	-	-	-				
Hydroxy methyl furfural	Aldehydes	-	-	-	-	-	6.66				
Nona-2(E).6(Z)-dienal	Aldehydes	-	-	-	2.36	-	-				
Tridecylaldehyde	Aldehydes	-	-	-	-	-	0.76				
Esters											
Propanoate <ethyl->	Esters	-	-	0.56	-	-	-				
Formate <amyl->	Esters	-	18.71	6.29	2.92	9.84	-				
Isovalerate <ethyl->	Esters	-	-	1.74	-	2.67	-				
Acetate <amyl->	Esters	-	1.86	-	-	-	-				
Butyrate <2-methyl-. ethyl->	Esters	-	-	0.92	-	1.27	-				
Myrtenyl acetate	Esters	-	-	-	0.51	-	-				
Capronate <ethyl->	Esters	-	-	1.13	-	1.71	-				
Formate <isoamyl->	Esters	-	-	-	-	0.79	-				
Hex-3(Z)-enyl butyrate	Esters	-	3.38	25.61	15.13	12.47	-				
Sabinene hydrate <cis->	Esters	0.41	-	-	0.84	-	4.77				
Benzoate <ethyl->	Esters	-	-	0.88	-	2.68	-				
Caproate <isoamyl->	Esters	-	-	-	0.59	-	-				

Butyrate <2-methylbutyl-. 2-methyl->	Esters	-	-	-	-	3.11	-				
Octanoate <ethyl->	Esters	-	-	-	-	1.59	-				
Phenylacetate <ethyl->	Esters	-	-	0.62	-	-	-				
Phenylacetate <ethyl->	Esters	-	-	-	-	0.77	-				
Isobornyl acetate	Esters	1.61	1.04	-	-	-	0.59				
Nonanoate <ethyl->	Esters	-	-	-	-	0.7	-				
Isobutyrate <isoamyl->	Esters	-	-	-	-	1.14	-				
Butyrate <isoamyl->	Esters	1.18	-	-	-	9.77	-				
Geranyl formate	Esters	-	-	-	-	-	0.35				
Hex-3(Z)-enyl acetate	Esters	0.47	-	-	-	-	-				
Phthalate <diethyl->	Esters	0.8	1.78	-	-	-	6.82				
Furans											
Furan <2.5-dimethyl->	Furans	-	-	-	-	-	1.21				
Furan <2-acetyl->	Furans	-	-	0.75	-	-	-				
Ketones											
Pyruvophenone	Ketones	-	-	1.06	-	-	14.68				
Ethyl n-propyl ketone	Ketones	0.5	3.93	0.79	-	-	-				
Ethyl vinyl ketone	Ketones	-	1.46	-	-	-	-				
Furan-3-one <2-methyl-. tetrahydro->	Ketones	-	-	0.68	-	-	-				
Butan-2-one <3-mercapto->	Ketones	-	-	-	-	-	4.51				
Amyl methyl ketone	Ketones	1.32	-	-	-	-	-				
Dihydrojasmone	Ketones	-	2.28	-	-	-	-				
Limonene oxide <cis->	Ketones	-	-	0.91	-	-	-				
Carvone <(E)-. dihydro->	Ketones	-	-	-	-	0.93	-				
Acetophenone	Ketones	-	-	-	1.53	0.74	-				
Heptyl methyl ketone	Ketones	1.18	-	-	-	-	-				

Isophorone	Ketones	-	-	-	0.5	-	4.5				
Heptylidene acetone	Ketones	-	-	-	-	-	4.76				
Limonene oxide <trans->	Ketones	-	-	-	0.41	-	-				
Pulegone	Ketones	-	-	-	0.4	-	-				
Acetophenone <2’-amino->	Ketones	-	-	-	0.74	-	-				
Organic acids											
Isobutyric acid	Organic acids	0.36	0.84	-	0.44	-	-				
Butyric acid	Organic acids	-	4.17	1.27	-	-	-				
Enanthic acid	Organic acids	2.85	-	-	-	-	-				
Isovaleric acid	Organic acids	1.24	1.49	-	-	-	-				
Caproic acid <2-ethyl->	Organic acids	-	-	-	0.44	-	-				
Benzoic acid	Organic acids	-	-	-	-	0.77	-				
Others											
Ethane <1.2-dimercapto->	Others	-	-	-	-	-	0.33				
Nonane	Others	17.68	7.22	3.95	7.39	8.27	20.3				
Benzene <amyl->	Others	-	-	-	0.77	-	-				
Furfural <5-methyl->	Others	1.94	-	-	-	-	-				
Cymene <para->	Others	-	-	-	6.11	-	-				
Ocimene <(Z)-.beta->	Others	-	-	-	0.46	-	-				
Dimethylstyrene <alpha-para->	Others	-	-	-	11.43	-	-				
Heptenal <4Z->	Others	-	-	2.49	0.85	-	-				
Undeca-1.3.5-triene	Others	-	-	0.54	-	1.31	-				
Geranyl nitrile	Others	0.48	-	-	-	-	-				
Geranyl nitrile	Others	-	-	-	1.71	-	-				

Table S2. Elemental concentrations of samples V1–V10.

Sample	Fe	Zn	Cu	Mn	Ni	B	Cd	Pb	Ca	Na	Mg	K	Al
V1	2.73	2.97	0.78	nd	0.23	2.34	0.16	nd	115.63	314.22	49.61	266.64	20.63
V2	2.91	102.14	0.55	0.59	0.18	2.27	0.09	nd	1250.91	215.23	48.77	255.73	22.55
V3	2.62	141.98	0.70	0.12	0.17	2.27	0.06	nd	61.57	231.28	37.79	292.27	20.35
V4	2.10	22.25	0.80	16.75	0.50	2.00	0.20	0.25	453.05	254.65	93.50	2162.00	16.20
V5	3.83	0.94	1.02	1.17	3.91	3.20	0.16	0.47	77.97	335.23	53.44	453.75	21.95
V6	2.58	9.88	0.65	3.92	0.15	2.92	0.08	nd	249.23	184.65	64.92	1055.38	19.31
V7	46,21	0,04	7,14	1,70	nd	1,54	0,08	nd	17,51	111,32	31,35	235,45	14,83
V8	54,79	0,77	9,11	0,27	nd	1,30	0,08	nd	52,18	91,00	22,36	131,47	66,08
V9	52,92	0,30	7,98	2,62	0,04	1,88	0,07	nd	22,30	80,87	38,22	298,12	23,74
V10	55,82	0,13	5,03	8,29	nd	0,54	0,08	nd	57,96	92,38	39,15	499,16	5,11

Nd: not detected
